# Minimizing the risk of graft failure after anterior cruciate ligament reconstruction in athletes. A narrative review of the current evidence

**DOI:** 10.1186/s40634-022-00461-3

**Published:** 2022-03-15

**Authors:** Giuseppe Gianluca Costa, Simone Perelli, Alberto Grassi, Arcangelo Russo, Stefano Zaffagnini, Juan Carlos Monllau

**Affiliations:** 1 Orthopaedic and Traumatologic Unit, Umberto I Hospital, Azienda Sanitaria Provinciale di Enna, C.da Ferrante, 94100 Enna, Italy; 2 Knee and Arthroscopy Unit, Institut Catalá de Traumatologia I Medicina de L’Esport (ICATME), Hospital Universitari Quiron Dexeus, Universitat Autonoma de Barcelona, Barcelona, Catalunya Spain; 3 Department of Surgery and Morphologic Science, Orthopaedic Surgery Service, Universitat Autonoma de Barcelona, Hospital Del Mar, Barcelona, Spain; 4 Clinica Ortopedica e Traumatologica II, IRCCS Istituto Ortopedico Rizzoli, Bologna, Italy

**Keywords:** Anterior cruciate ligament reconstruction, Failure, Prevention, Surgical technique, Return to sport, Athletes

## Abstract

Anterior cruciate ligament (ACL) tear is one of the most common sport-related injuries and the request for ACL reconstructions is increasing nowadays. Unfortunately, ACL graft failures are reported in up to 34.2% in athletes, representing a traumatic and career-threatening event. It can be convenient to understand the various risk factors for ACL failure, in order to properly inform the patients about the expected outcomes and to minimize the chance of poor results. In literature, a multitude of studies have been performed on the failure risks after ACL reconstruction, but the huge amount of data may generate much confusion.

The aim of this review is to resume the data collected from literature on the risk of graft failure after ACL reconstruction in athletes, focusing on the following three key points: individuate the predisposing factors to ACL reconstruction failure, analyze surgical aspects which may have significant impact on outcomes, highlight the current criteria regarding safe return to sport after ACL reconstruction.

## Introduction

Anterior cruciate ligament (ACL) tear is one of the most common sport-related injuries, involving about 3% of amateur athletes every year, and up to 15% of elite athletes per year [87]. The international literature unanimously agrees on the importance of performing surgical reconstruction in active patients, in order to properly restore the joint kinematics, preserve the intraarticular knee structures and increase the likelihood to resume preinjury sport activities [50, 58, 101].

Despite the recent advances in arthroscopic equipment, understanding knee biomechanics and surgical techniques, unfortunately ACL reconstruction is not always successful, but a significant number of patients (10% to 15%) [116] reports unsatisfactory outcomes. Previous systematic reviews reported only 60% of amateur athletes [5] and 83% of elite athletes [62] returned to their preinjury sport level after ACL reconstruction. Graft failure is one of the main determinants of outcomes, representing a traumatic and career-threatening event in athletes. In a meta-analysis involving 1272 elite athletes, the pooled failure rate was estimated in 5.2% (range 2.8% - 19.3%) [62], but this rate has been shown to grow up to 34.2% when including high-risk cohorts like younger athletes [142]. The outcomes after revision ACL reconstructions are shown not as good as primary reconstructions, in terms of functional scores, rotatory stability, and risk of developing knee osteoarthritis [39, 89].

It can be convenient to understand the multiple risk factors for ACL graft failure, in order to properly inform the patients about the expected outcomes and to minimize the chance of poor results. In literature, a multitude of studies have been performed on the risk factors of failure after ACL reconstruction, but the huge amount of data may generate conflicting evidence. A comprehensive analysis of this information may support those who want to approach this issue with an evidence-based methodology.

The aim of the current review is to examine data collected from literature about the risk of graft failure after ACL reconstruction in athletes, focusing on the following three key points: (1) identify the predisposing factors to ACL reconstruction failure, (2) analyze surgical aspects which may have significant impact on outcomes, and (3) highlight the current criteria regarding safe return to sport after ACL reconstruction.

### Predisposing factors

Identifying predisposing factors for graft failures can represent a successful approach for several reasons. First, patients can be better informed about the chances of failure after an ACL reconstruction. Secondly, this information can be used for developing strategies to modify manipulable factors and, therefore, reduce the risk of failure. For convenience, predisposing factors will be classified as demographic, anatomical and environmental factors.

### Demographic factors

Age is universally recognized as independent factor affecting risk for ACL graft failure. In a recent systematic review including 33 studies from 4 different national registries [111], young age was reported as independent risk factor for revision ACL surgery in all registries. Patients aged under 20 years were found to have a risk three times higher than patients over 20 years old, four times higher when compared to patients over 30 years old and nearly eight times higher than patients aged 40 years or older [111]. In another prospective analysis of 2488 primary ACL reconstructions, the authors found that the likelihood of failure decreased by 9% for each increasing year of patients’ age [58]. One of the reasons may be the higher activity level in younger patients, which is shown to significantly affect the risk of reinjury [55]. In addition, Nakanishi et al. [98] evaluated the anteroposterior stability with arthrometric testing of two groups of patients undergoing ACL reconstruction and found that younger group had a greater tendency for residual knee joint laxity. This joint laxity could alter dynamics of lower limbs motions and predispose to failure [59].

If the evidence for age can be defined as high, the same cannot be stated for patient gender as significant factor. Some registry studies demonstrated a higher risk for ACL revision in male patients [16, 130], whereas other registry data deny this finding, reporting a greater risk in female patients [2]. In addition, several other similar studies failed to demonstrate a statistically significant relationship between patient gender and ACL revision [55, 73, 111]. A recent meta-analysis including 135 articles showed that graft failure rates did not differ significantly between sexes [132]. However, the inclusion of a such impressive number of studies is not immune from plausible confounders, such as differences in activity level or age distribution of the groups. The anthropometric sex-based differences, as well as sex hormonal influence deserve further investigation with higher methodological quality.

### Anatomical factors

Several anatomical factors have been directly correlated with increased rate of ACL injury but there is poor evidence about the correlation of such anatomical patterns and risk of graft failure after ACL reconstruction. This is especially true for the body mass index (BMI). Two registry studies on 12,643 patients [108] and 21,304 patients [81], respectively, found a lower risk for ACL revision in patients with higher BMI. In contrast, a cohort study on 30,747 patients from the Norwegian and the Swedish National Knee Ligament Registries reported an increased risk for ACL revision within 2 years both in male and female patients with higher BMI [125]. However, this risk was higher especially for those patients with BMI between 25 and 30, whereas it significantly decreased in patients with a BMI > 30. The different neuromuscular control as well as the patients’ level of participation in sport activity might affect the validity of this line of research, but on the other hand, can represent a convincing explanation of such findings.

Another interesting chapter is the relationship between bony knee anatomy and risk for graft failure.

**Fig. 1:**
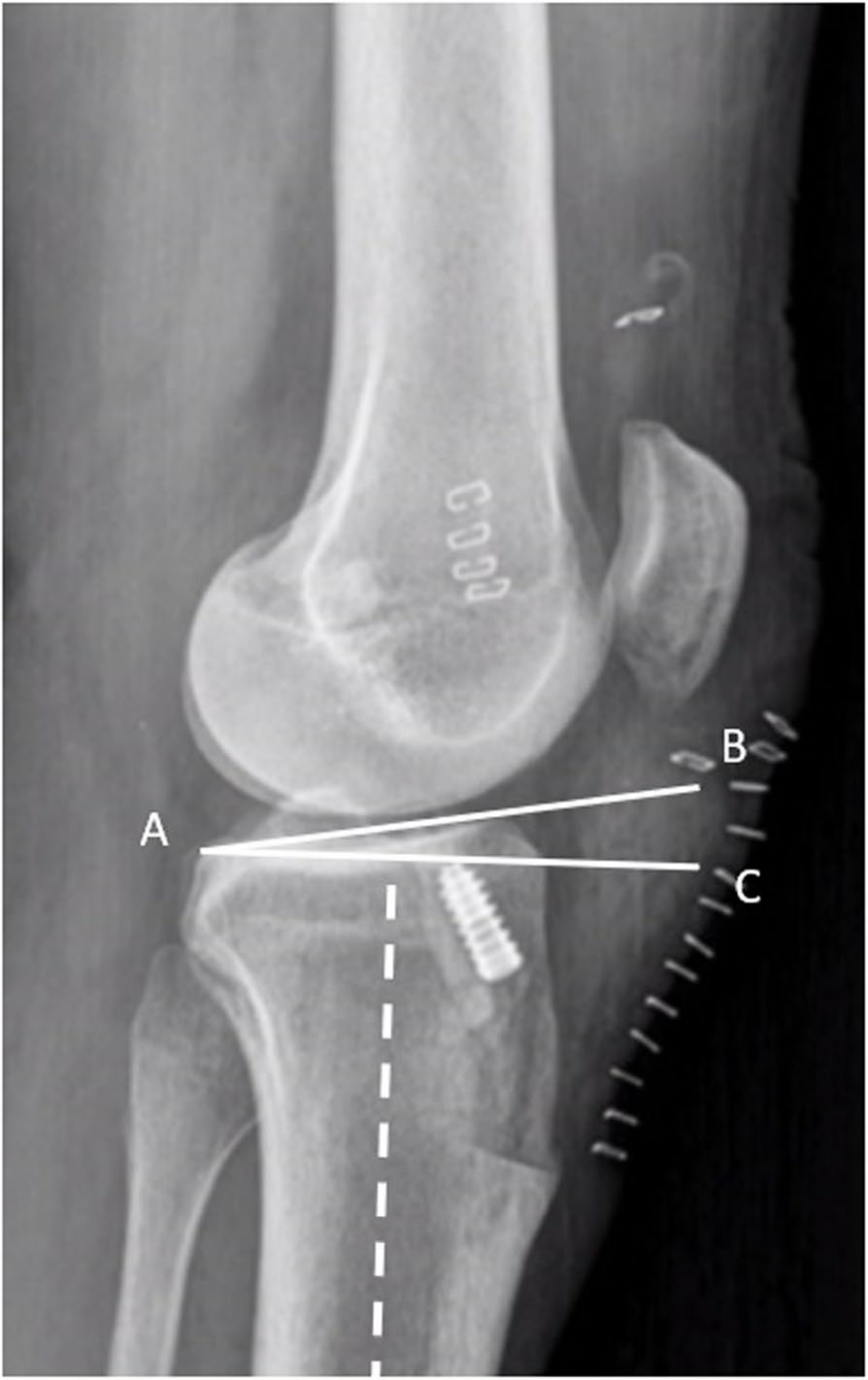
The lateral posterior tibial slope, that is the angle between the tangential line to the surface of the lateral tibial plateau (line AB) and the perpendicular to the tibial axis (line AC)

Several anatomical features have been invoked over the years, including the lateral tibial slope, the intercondylar notch, the lateral femoral condylar offset, the alpha angle (that is the angle between the longitudinal axis of the femur and the Blumensaat line), the lateral femoral notch sign depth, the tibial eminence size, the lateral tibial plateau diameter, and many others [9]. All these bony morphologic features have been advocated as predisposing factors for native ACL rupture, but their effect on the risk of graft failure remains indefinite [42]. Among these, the lateral tibial slope (Fig. 1) has gained more attention among physicians in the last few years. A study on human cadavers reported that an increased lateral tibial slope was significantly associated with anterior tibial acceleration and ACL strain during simulated jump landing task [11]. Several studies found a significantly higher value of lateral tibial slope among patients with a failed ACL reconstruction, when compared to patients who did not experience graft failure after reconstruction [19, 42, 45, 54, 115, 148]. Considering this background, some authors advocated a combined closing-wedge anterior high tibial osteotomy in cases of multiple ACL reconstruction failures in the absence of technical errors and with a radiographic lateral tibial slope > 12° [72].

The evidence regarding the effect of the remaining anatomical variables on the risk of ACL graft failure is poor. This is also true for the intercondylar notch, discussed as early as 1980s [9]. Theoretically, a small intercondylar notch could create wear of the graft on the lateral femoral condyle during knee extension and internal rotation movements [36]. However, some recent studies on human cadaveric knees [53] and post-operative imaging analysis [42, 52, 144] demonstrated that, if the graft is correctly placed, impingement should not occur, and therefore the risk for failure is not increased.

### Environmental factors

Environmental factors include both extrinsic aspects to athlete (such as type of resumed sport, playing surface, footwear etc.) and biomechanical aspects of playing actions which may predispose to graft retear. Since all those are modifiable factors, large research efforts have been made to create preventive programs focused on these issues [4].

Participation in pivoting and hard cutting sports is a well-known predictor of further graft tear after ACL reconstruction. It is estimated a four-times increased risk of knee reinjury among athletes of such sports activities [44]. However, modifying activity level is not always suitable, because intent to return to high level sports is often the main reason why a patient with an ACL tear undergoes arthroscopic reconstruction. Therefore, specific sessions including plyometric exercise, neuromuscular reeducation, balance and strength training have been advocated to prevent knee reinjuries [44, 99]. For instance, dynamic valgus collapse during weightbearing activities (such as cutting, landing or changing direction movements) was found to be predictor of non-contact ACL injury [43]. This may be due to specific muscular weakness (hip abductors, knee flexors) as well as some predisposing anatomical features, such as increased femoral anteversion or external tibial torsion [120]. A proper balance between quadriceps and hamstring activation is critical to not overload the knee during the landing after a jump. Specifically, hamstring recruitment reduces ACL loads at landing [143] and may help to provide dynamic knee stability by resisting anterior tibial translation and rotations [67]. Based on this, several interventional studies describing specific neuromuscular and plyometric prevention programs demonstrated a significant reduction in the incidence of ACL injuries [4, 44, 99].

### Surgical procedure

Graft failure after ACL reconstruction may result from any combination of technical errors, biologic causes and traumatic events. Historically, technical errors have been considered the most important cause of graft failure [139]. A recent systematic review [139] conducted on 3567 failures identified technical errors as one of the most common causes of failure, preceded only by traumatic events. Similarly, Karmath et al. [56] reviewed the literature regarding outcomes after ACL reconstruction and reported that technical errors (e.g., improper tunnel placement, inadequate ACL graft, insufficient graft tensioning and failure to recognize concomitant laxity) accounted for 22% to 79% of failure cases. Therefore, it should not be surprising that technical aspects of ACL reconstruction have always been a major focus for scientific investigation. With the aim to provide an exhaustive synthesis of the huge amount of data published in the literature, this section will focus on the proper management of concomitant lesions, the outcomes related to different graft types and the evidence about surgical technique.

### Concomitant lesions management

When planning an ACL reconstruction, an assessment of the other ligaments as well as intra-articular structures of the knee should not be omitted. Associated lesions can compromise the graft function due to residual instability. It is estimated that about 15% of ACL reconstruction failures can be result of a missing diagnosis of associated ligament or meniscus lesion at time of surgery [34, 116].

One of the most discussed issues about this topic is the protective effect of the anterolateral ligament (ALL) on the ACL graft function. This interest is fueled by the common finding of residual pivot-shift phenomenon after ACL reconstruction, which is estimated in up to 25% of cases regardless the chosen graft [127]. Furthermore, persisting rotational instability has been shown to be a risk factor for recurrent injuries and ACL failure [127]. Anterior translation, internal rotation, and pivot shift was found to be better restored with combined ACL/ALL reconstruction than with ACL reconstruction alone in several biomechanical studies [60]. Lateral extra-articular tenodesis (LET) procedures have also been found effective in reducing tibial internal rotation and intra-articular ACL graft force [122], although the risk of knee overconstraint has been reported [122]. This can be reduced if the graft is attached proximal to the lateral epicondyle and courses deep to the fibular collateral ligament [122].

Such biomechanical findings also result in clinical evidence of reduced risk of graft failure [93]. A recent meta-analysis of 20 randomized and nonrandomized controlled trials found that the rate of graft failure was two-to-four times lower in the ACL plus ALL reconstruction/LET group than in the isolated ACL reconstruction group, regardless the adopted technique or the surgical timing [94]. In contrast to ALL reconstruction techniques, patients who underwent LET combined with ACL reconstruction were found to be more prone to suffer of knee stiffness and adverse events [95]. In another meta-analysis including 7 randomized controlled trials, graft failure rate was 3 times less likely in patients who underwent an ACL reconstruction with LET when compared to patients with isolated ACL reconstruction [104].

Based on such evidence, international literature supports such additional procedures in high-risk patients. Indications include patients with high-grade pivot shift, concomitant Segond fractures, high-level athletes participating in pivoting sports and in ACL revision settings [127].

Medial collateral ligament (MCL) injury is frequently associated to ACL tears [38], as a result of the valgus stress component of a typical ACL trauma. ACL and MCL play a concomitant role in maintaining anteromedial knee stability [141]. Several cadaveric studies demonstrated that ACL strain is increased after sectioning MCL, when applying a valgus stress or an intra-rotation movement of the tibia [8, 141]. In addition, combined MCL and ACL sectioning increases anterior knee laxity greater than isolated ACL sectioning [80]. Despite these findings, the treatment of combined ACL and MCL tears is still controversial. Most authors support the conservative management of the MCL injury, especially in acute settings and low-grade injuries [12, 38]. A “wait and see” approach is recommended by some authors also in high-grade MCL tears [38]. However, a recent study from the Swedish National Knee Ligament Registry highlighted a higher risk of ACL revision in patients with ACL reconstruction and non-surgically treated MCL injuries compared to isolated ACL reconstructions [131]. When a repair or reconstruction of concomitant MCL injuries was performed, this risk was comparable to isolated ACL reconstructions [131]. These findings encourage the authors supporting early MCL repair or reconstruction [27] because ACL insufficiency might adversely affect the MCL process healing [145]. On the other hand, delayed ACL reconstructions have been related to better functional outcomes with earlier motion recovery [90]. MCL surgical treatment should be considered in patients with severe valgus alignment, entrapment over the pes anserinus tendon (Stener-like lesion), large bony avulsions and persistent instability after ACL reconstruction [27, 90].

The posterolateral corner (PLC) of the knee is another important issue of academic interest, because of an evolving appreciation for its biomechanical relationship with the ACL. PLC injuries are commonly associated to cruciate ligaments tears, occurring in isolation in only 28% of cases [25]. Specifically, 7.4% - 13.9% of patients with ACL injury have a concomitant PLC injury [64]. Biomechanical data demonstrated a significant increase in force on the ACL in PLC-deficient knee, when applying a varus moment or a combined varus-internal rotation moment to the knee joint [63, 109], as well as during simulated gait and squatting [57]. In addition, Plaweski et al. [109] found that an ACL reconstruction was not enough to prevent varus and external rotation displacement in the setting of ACL-PLC deficient knee; a return to native kinematics was achieved only after adding a reconstruction of PLC static structures. Despite such promises, the role of PLC on the risk of ACL failure has not been adequately investigated. In one registry study, a concomitant PLC injury would appear to not affect the risk of ACL failure, whatever the treatment is [131]. However, this analysis was impaired by the small size of the study groups, which limits the relevance of such findings.

**Fig. 2:**
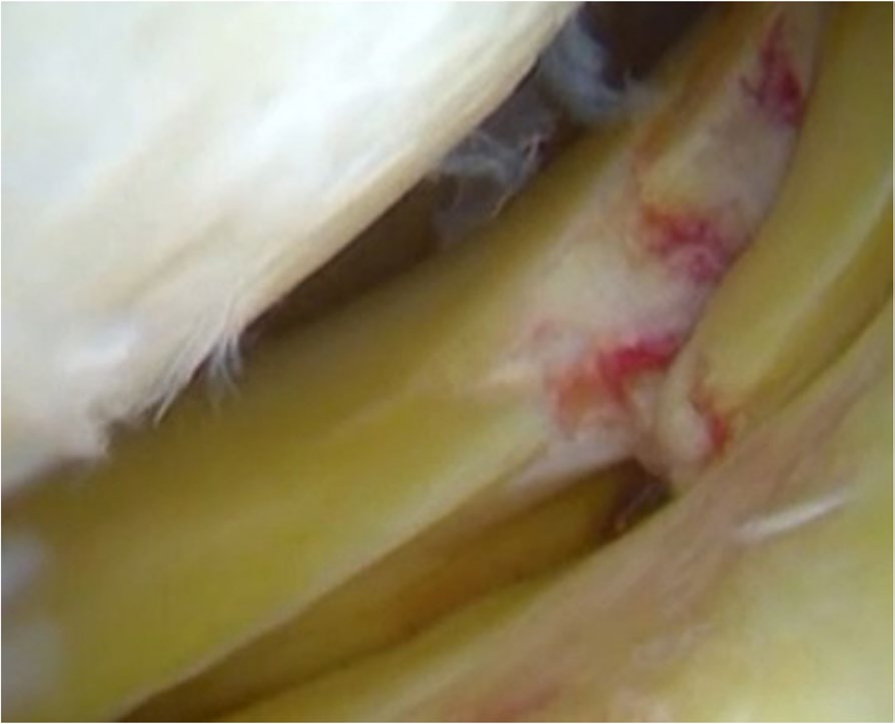
Lateral posterior meniscus root lesion, which are reported to significantly increase the anterior tibial subluxation of the lateral compartment in patients with ACL injuries

**Fig. 3:**
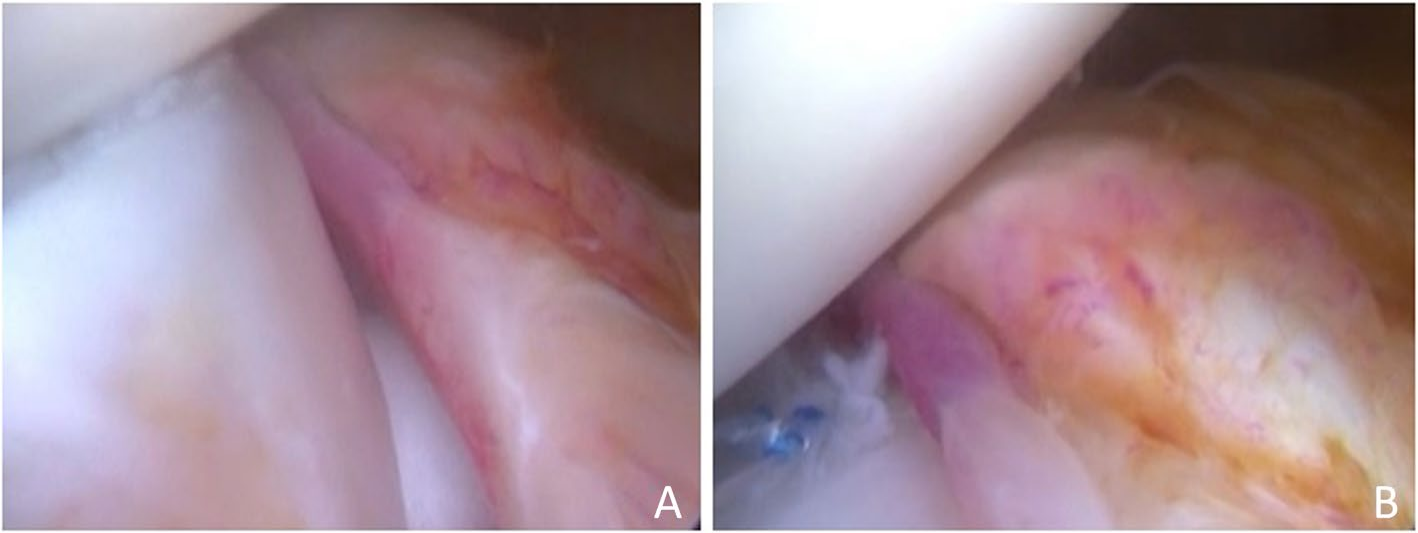
The ramp lesion, defined as posteromedial meniscocapsular disjunction and visualized with trans-notch view (**A**). The meniscocapsular repair with all-inside technique helps to restore native knee kinematics in concomitant ACL tears (**B**)

At last but not least, the biomechanical influence of the menisci on knee stability must not be overlooked. It is well known that the medial and lateral menisci contribute to knee stability, acting as secondary restraints for anterior and rotatory tibial displacement [41, 46, 94]. Meniscus repair would seem to restore knee stability comparable to ACL-reconstructed knees with intact menisci [46]. These findings also apply to meniscus posterior root lesions (MPRL) [117, 153]. Lateral MPRLs (Fig. 2) were reported to increase anterior tibial subluxation of the lateral compartment in patients with ACL injuries [153]. Similarly, medial MPRLs were found to significantly increase ACL graft loads over the intact state, while root repair restored the function of the medial meniscus as a secondary stabilizer [117]. Finally, a ramp lesion in an ACL-deficient knee has also been shown to increase anterior tibial translation and external rotational laxities [95, 129]. This aberrant laxity cannot be completely restored after ACL reconstruction alone but with combined posterior menisco-capsular repair (Fig. 3) [96]. Nevertheless, there is poor clinical evidence regarding increased risk of graft failure following meniscal loss. Only one study identified medial or lateral meniscus deficiency as significant factor for predicting graft failure [107], while several other studies did not detect significant difference between isolated ACL reconstruction and ACL reconstruction combined with medial and/or lateral meniscectomy [3, 111, 149]. However, meniscectomy has been clearly recognized as a risk factor for delayed return to sport [3] and career shortening in athletes [3, 13, 100]. As a result, meniscus repair should be considered even in athletes.

### Graft choice

Graft choice has always been one of the most critical topics for discussion. The “ideal graft” used for surgical ACL reconstruction should recreate, as far as possible, the biomechanical properties of the native ligament, providing rapid biological integration and reducing recovery.

Historically, autologous grafts have been considered as the first-choice graft [6], since allografts and synthetic grafts have been proved to be inferior in terms of failure rates, clinical scores, and knee stability [23, 32, 48, 49, 111], especially among younger patients [23, 48]. Actually, bone-patellar tendon-bone (BPTB) is the overwhelming favorite over hamstring grafts in athletic population [40, 82], although quadriceps tendon (QT) has renewed interest among physicians as a potential alternative [6].

The available evidence in literature is mixed on which graft type is associated with a higher risk of graft failure and revision ACL reconstruction. In a systematic review conducted in 2017 and including all available meta-analyses focused on comparison between BPTB and hamstring grafts [119], the authors found that 10 out of 13 meta-analyses failed to demonstrate statistically significance between the two groups regarding the graft failure rate. More recently, a systematic review exclusively involving athletic population [26] demonstrated similar failure rates between BPTB (2.2%) and hamstring autografts (2.5%), but a trend for higher return to sport rates was found in athletes with BPTB autografts (81%) when compared with hamstring autografts (70.6%). The association between graft choice and the rate of revision has also been investigated in several registry studies [111]. In a systematic review collecting data from 11 registry studies [111], a statistically significant lower revision risk in favour of BPTB in comparison to hamstring grafts was reported in nine out of eleven studies. This reduced risk seemed to be slightly more pronounced for younger patients and for athletes involved in pivoting activities, such as soccer, team handball, and alpine activities [111]. However, when interpreting such data, the influence of some confounding bias needs to be considered, firstly the role of surgery volume. It has been previously demonstrated that lower volume surgeons in lower volume hospital prefer hamstrings over BPTB autografts [51]. Lower volume sites have been associated with more patient-reported subjective failures of ACL reconstruction [84] and subsequent revision surgeries [79]. Finally, a recent meta-analysis [7] pointed out a higher incidence of deep infections after ACL reconstruction with hamstring autografts compared with BPTB autografts. Although it is an unusual complication, it should deserve particular consideration because of the potentially deleterious effects on graft function, knee joint and athletes’ career, taking into account that professional athletes are defined as a risk category [128].

Outcomes of QT graft were evaluated in three recent meta-analyses [93, 102, 113]. Riaz et al. [113] firstly demonstrates comparable survival rates and joint stability when BPTB and QT grafts are used, but with fewer adverse donor site symptoms using QT grafts. Later, such findings were confirmed by Mouarbes et al. [93] in a systematic review of 2856 patients, reporting QT grafts have comparable graft survival rate to BPTB and hamstring, with less harvest site pain than BPTB autograft and better functional outcome scores than hamstring autograft. Nyland et al. [102] found that QT autografts had lower failure rates than hamstring autografts, but difference was overturned when a suspensory femoral fixation was used in hamstring group. This led to the suggestion that graft fixation is also an important aspect of surgical failure. Surprisingly, a recent registry study from the Danish Knee Ligament Registry [75] reported a statistically significant higher risk of failure for QT graft (4.7%) in comparison to both BPTB (1.5%) and hamstrings graft (2.3%) at 2-year follow up. However, the smaller samples size, the lower patients’ age and the higher incidence of concomitant meniscus and cartilage injuries in the QT cohort represent a relevant bias. In addition to this, the same authors revealed the considerable influence of the learning curve on the outcomes of ACL reconstruction with QT, since revision rates dropped to 0.8–2.0% when low volume clinics with less than 100 procedures per years were excluded [74].

As it can be deduced from all these data, there is no evidence regarding superiority of one autograft over the others. Each graft presents both advantages and issues that need to be considered. For example, BPTB autografts have some well-documented morbidities including postoperative anterior knee pain, difficulty kneeling, and the risk of extension deficit [93, 113, 119]. On the other hand, proponents of hamstring autografts reported less donor-site morbidity, but increased weakness in hip extension and terminal knee flexion, as well as variable outcomes related to graft size and length [93]. If the hamstring graft size is equal or less than 8 mm, the risk of failure was found to increase by 6.8 times [22]. Despite some fascinating biomechanical promises, QT graft remains the least studied and least used autograft [88]. The lack of long-term trials makes the QT a difficult choice for surgeons, who prefer grafts that have been shown to be safe and clinically efficient in the long term. As a result of what has been said, it is reasonable to make an individual graft choice, based on patient’s expectation, body characteristics and kind of sport resumed.

### Surgical technique

Proper positioning of the ACL graft has been proven to be of utmost importance to reduce risk of graft failure [92, 139]. Non-anatomic graft positions create not physiological intra-articular force vectors, which may affect graft longevity. For instance, a graft that is placed too posterior or too low in the femoral condyle edge is subjected to higher tension during knee extension [83]. Conversely, a high and anterior position produces a longer and more “vertical” graft, which results in increased anterior tibial translation [1] and increased rotational laxity [66, 150]. In addition to the above, graft positioning also influences the risk of graft impingement [105]. This may impact not only knee motion, but also risk of graft failure [47]. According to the above, it is recommendable to place femoral and tibial tunnels as close to the native ACL footprints as possible, in order to reproduce more closely the biomechanical properties of the native ACL [10, 92, 150].

**Fig. 4:**
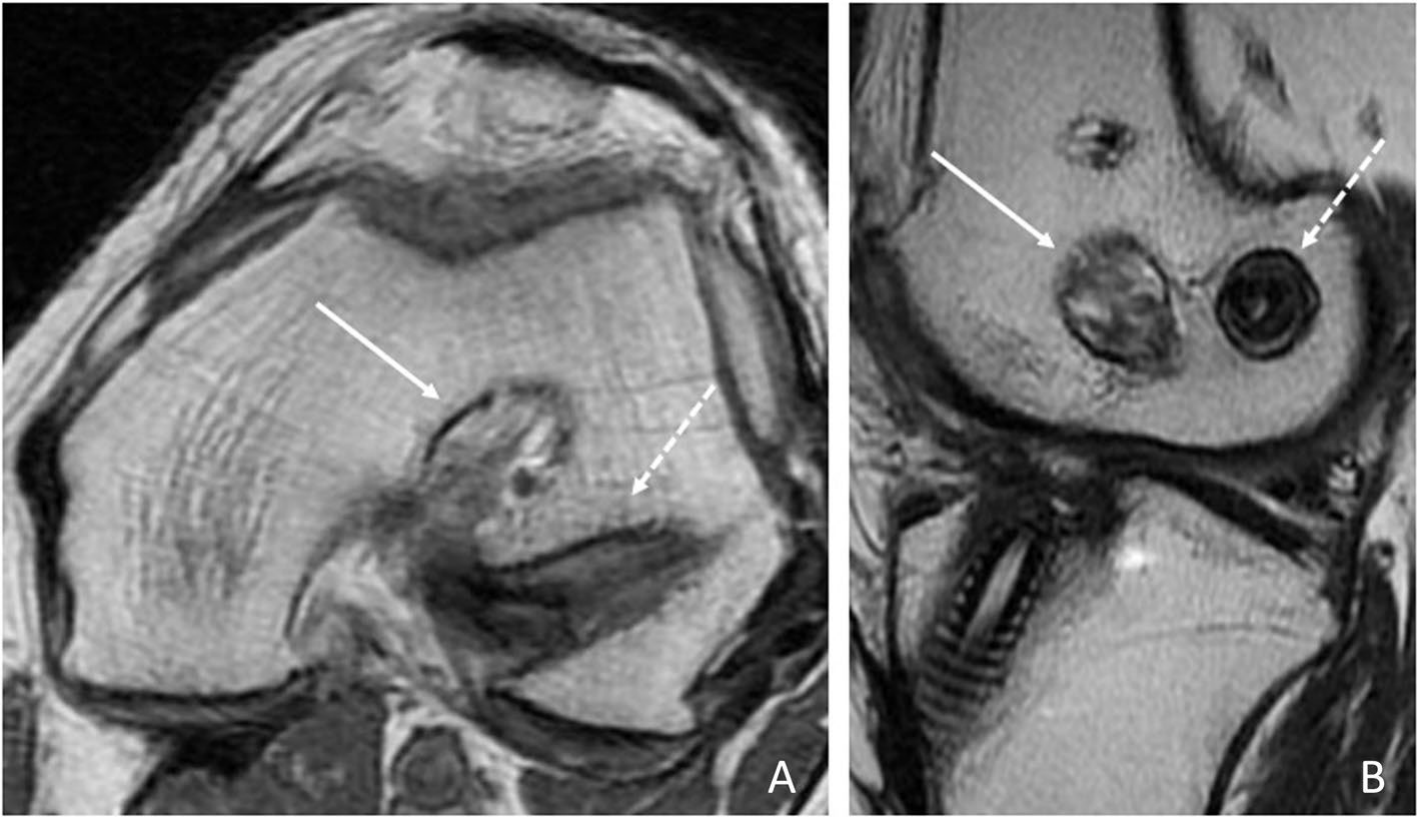
MRI axial (**A**) and sagittal (**B**) scans showing the case of a 24-year-old patient undergoing a previous non-anatomic ACL reconstruction with a transtibial technique (white arrow) and the new anatomic femoral tunnel placement (dotted line) using an anteromedial portal technique

The transtibial technique makes it more difficult to address accurately and reliably the femoral ACL footprint [65]. As a result, several physicians support tibial tunnel-independent methods for femoral tunnel placement, which have been proven to provide a more anatomic positioning of both the tibial and femoral tunnels (Fig. 4) [65, 114]. In accordance with such biomechanical evidence, international literature demonstrated that tibial tunnel-independent techniques result in better knee stability and functional outcomes [18, 78, 90, 114]. Accordingly, these techniques should better protect the knee from further joint injuries [30] and osteoarthritis development [20]. This was confirmed by a recent meta-analysis including a total of 1546 patients [20], but such findings are affected by the lack of a more in-depth analysis of concomitant meniscal injuries, thus representing a relevant bias that may have influenced the observed rates of osteoarthritis development. Despite this, there is no evidence of lower subjective outcomes scores [18, 91] or increased graft failure rates [24, 78, 114] with the transtibial technique. In addition to this, there are several registry studies showing higher graft revision rates with the anteromedial portal technique [111]. Some authors argued that an anatomic reconstructed ACL graft is subjected to greater force than non-anatomic high placement of ACL graft [147]. Moreover, the tibial tunnel-independent techniques have shown to produce a higher graft bending angle than the transtibial technique [133]. This angle was demonstrated to significantly affect the graft signal and femoral tunnel diameter at 12 months [68], although the clinical relevance of such finding is unclear, because functional outcomes, arthrometric data and subjective scores seem to not be related [68]. Finally, the learning curve of the more demanding “tibial tunnel-independent” has been advocated as part of the explanation of such findings [112], although the anteromedial portal method has been reported as the most used technique for femoral tunnel drilling throughout the world [88]. With improved understanding of the anatomy and biomechanics of the ACL, the transtibial approach has been modified to achieve a more anatomic femoral tunnel placement. The modified transtibial technique showed superior outcomes than conventional transtibial approach and comparable with the anteromedial portal technique in terms of clinical scores, negative rates of the Lachman and the pivot-shift test, and return-to-sport level [69]. Future studies are needed to determine the long-term benefits with the modified transtibial in terms of graft failure rates.

In addition to the above, alternative techniques have been supported aiming to improve outcomes and graft survival. Further developing the concept of anatomical ACL reconstruction, the double-bundle reconstruction has been proposed to replicate the anteromedial and the posterolateral bundles. Several biomechanical studies supported this technique, demonstrating improved anteroposterior and rotational knee stability [103]. However, this promising background resulted in a clinical small difference in terms of joint stability [17, 28, 29, 70, 71, 86], but not in functional and subjective scores [17, 28, 29, 71, 86, 103], as well as in terms of failure rate [17, 28, 71, 86], since only one meta-analysis demonstrated a lower risk of graft failures with double bundle ACL reconstruction [70].

More recently, there is an increasing interest in replacing conventional round tunnels with tunnel shapes that resemble more closely the original ACL footprints. The basic principle of these techniques comes from some anatomic studies describing the ACL as a flat, “ribbon-like” structure, with a thin, oval-shaped insertion on the femur and a C-shaped tibial insertion [121, 123, 124]. The proposed advantages are both biomechanical with increased rotational stability [151], and biological due to increased bone-tendon contact and decreased distance to the central part of the graft [152]. Despite preliminary promising data, clinical benefits over conventional ACL reconstruction techniques have yet to be demonstrated with high-quality methodology studies.

In the last few years, a renewed interest in ACL repair has arisen. This is not surprising because of the new paradigm shift toward restoring native anatomic features and the improved knowledge of orthobiologics. This can be convenient in particular subsets of patients, such as skeletally immature patients with acute, proximal ACL tears [136]. Biologically enhanced arthroscopic ACL repair may help to improve anteroposterior knee stability and patient-reported outcomes, although there is no evidence of reduced rate of surgical failure [15]. However, historical reports showing unacceptable high failure rates at long-term follow-up [134] prevent in recommending this procedure at present in patients with high functional requests, such as athletes.

### Return to sport

One of the greatest challenges for clinicians is to return the injured athlete back to sport as quickly as possible, but at the same time not exposing the affected knee to excessively high reinjury risks. Unfortunately, the risk of sustaining a second ACL injury is highest during the early period after return to sport (RTS), especially during the first year after the index reconstruction [4, 118]. As a result, definition of rigorous and well-coded RTS criteria has always been a main research focus.

Time after ACL reconstruction is the most used criterion to assess RTS readiness [14]. In a recent scoping review of 209 studies [118], time to RTS was reported as criterion in 85% of included studies and represented the sole criterion to give athlete the all-clear to RTS in 42% of studies. It goes without saying that time is a crucial variable for proper graft integration and maturation [85]. Historically, six months for contact sports were considered a good compromise [14]. Recently, this axiom has been questioned. The Delaware-Oslo ACL cohort study found that delaying RTS at 9 months after ACL reconstruction may reduce reinjury risk by 84% [45]. Specifically, the reinjury rate was reduced by 51% for every month delay for up to 9 months, beyond which no further risk reduction was observed. Furthermore, some authors even supported delay of RTS until two years, calling into question biological and rehabilitative argumentations [97].

However, it is obvious that time alone is not sufficient for determining readiness for sports resumption [14, 44]. Some authors proposed to focus instead on graft maturation and functionality [33]. Histologic analysis of biopsy graft specimens during second-look arthroscopy is considered the gold standard to determine graft maturity [21]. Nevertheless, this method is invasive and, therefore, not feasible for clinical follow-up. Magnetic resonance imaging (MRI) may be useful for indirect monitoring of graft “ligamentization” process, as incomplete graft maturation is related to a hyper-intense graft signal on MRI [137]. However, no evident correlation was found between signal intensity and knee stability outcome scores [137]. Therefore, a routine MRI assessment of graft maturity does not provide solid insights for RTS. Ideally, the information gained through MRI assessment should be combined with laxity measurements, to follow the graft evolution and early detect potential abnormalities (graft elongation, iterative rupture, contralateral rupture, etc.). Both anteroposterior and rotatory stability is required to safely RTS. Therefore, non-invasive devices for anteroposterior stability and pivot shift assessment have been developed in the last years, both to diagnose ACL injury and to detect residual laxity after ACL reconstruction [77, 110]. Such technologies could represent a potential aid in the follow-up evaluation of patients undergoing ACL reconstruction and in the RTS decision algorithm. An anteroposterior side-to-side difference < 5 mm is unanimously accepted as threshold for defining a knee as sufficiently stable [33, 106]. On the other hand, a standardized quantification of knee rotatory laxity is still lacking [77]. The variability of the pivot shift outcome, for both displacements and accelerations, depends on how the tester is performing the maneuver itself, in terms of both the magnitudes of the applied loads and the speed with which the limb is moved [77]. Furthermore, clinical studies reported knee laxity measurements at a specific time point after ACL reconstruction. Thus, little is known about the evolution of knee laxity over the months. These conclusions are still difficult to generalize, due to the diversity of such variables as surgical techniques, graft types, fixations devices, associated injuries and measurement techniques.

Muscular strength recovery is another fundamental requirement before RTS. Above all, isokinetic testing measures have been reported for proper evaluation of quadriceps and hamstring strength [14, 138]. In addition to this, functional and performance test have been supported to enhance their predictive value [138]. Among these, hop tests have become the mainstay of performance tests prior to returning the athlete to sport, with the numerous variations which have been added over the years [14, 138]. Limb symmetry index (LSI) has been widely adopted as a reliable measurement outcome. A LSI ≥ 90% is supported before RTS [14], although some authors recommended an LSI ≥ 100% for higher impact sports athletes [135]. However, there are some concerns regarding the use of the uninvolved limb as a reference for the involved limb. LSI may overestimate knee function since the resulting reduction in sports participation following ACL injury leads to bilateral muscle strength deficits [31]. Therefore, LSI could not be specific enough to indicate the athlete has reached the preinjury level. For this purpose, some authors proposed to consider the estimated pre-injury capacity (EPIC), that is obtained by comparing the involved-limb measures to uninvolved limb measures before ACL reconstruction [140]. Wellsandt et al. [140] demonstrated that 90% EPIC levels were more sensitive than 90% LSI levels at assessing the risk of ACL re-injuries. On the other hand, the preinjury level may be not sufficient for safe sports participation and performance. Furthermore, the outcome measure of hop tests and isokinetic tests is strictly quantitative in nature, while outcomes related to the quality of movement are not captured [146]. In order to solve those issues, Padua et al. [106] proposed a clinical assessment tool for qualitative analysis of aberrant movements during a standardized jump-landing test. Gokeler et al. [35] applied this score in a cohort of 28 patients who underwent ACL reconstruction. By doing so, the authors were able to detect 30% of patients with aberrant movements which may predispose to increased risk of ACL reinjury [35]. Moreover, the quality of movement is significantly affected by fatigue [35, 106]. Thus, repetitive testing is encouraged for proper evaluation of ACL-reconstructed knee kinematics. The evolving research has made available new technologies for more refined kinematics analysis, including gait analysis, force-plates, electromyography and virtual immersive analysis [61]. However preliminary findings need to be confirmed with high methodological quality studies.

Psychological aspect is another matter that should be considered before clear the athlete back to sport. The injury and time spent out of match can impair athletes’ motivation, that has been shown to play a key role for returning to pre-injury sport level [126]. Patient’s perception of symptoms, function and activity can be reliably estimated with various patient-reported outcome measures (PROMs). However, it is debated in literature whether PROMs may reliably predict risk of ACL reinjury. Granan et al. [37] observed an increased risk of graft failure in patients who had poor Quality of Life subscale of KOOS at 2 years after index ACL reconstruction. Similarly, Logerstedt et al. [76] reported that patients who scored poorly on the IKDC were over four times more likely to fail the RTS tests. On the other hand, nearly 50% of the athletes with good scores overestimated their recovery [76].

From the foregoing, it is clear that the decision to allow RTS after ACL reconstruction solely based on one single criterion (time, strength recovery, functional test, PROMs) cannot be adequate. An all-around evaluation including biological, kinematic and psychological aspects is strongly recommended. Therefore, battery of tests including multiple measurements should be performed, instead of one single assessment at the hypothetical end of rehabilitative process. A stepwise evaluation process during the entire rehabilitation process is thus indicated.

## Conclusion

This review collected and summarized a large body of research addressing the risk of ACL failure. The current evidence available in literature shows that surgical technique represents a key factor, but this aspect alone is insufficient to ensure long-term graft survivorship. Instead, a careful preoperative evaluation is necessary, in order to detect any predisposing factor which may increase risk of graft failure, and therefore address it where possible. Similarly, the post-operative rehabilitation phase needs a global stepwise evaluation and should be managed by a specialized sport-traumatology team. Final RTS clearance decisions should positively balance the athlete’s desire to savor the playing field with the risk of graft reinjury.

## Abbreviations

ACL: Anterior cruciate ligament
BMI: Body mass index
ALL: Anterolateral ligament
LET: Lateral extra-articular tenodesis
MCL: Medial collateral ligament
PLC: Posterolateral corner
LMPR: Lateral meniscus posterior root
BPTB: Bone-patellar tendon-bone
QT: Quadriceps tendon
RTS: Return to sport
MRI: Magnetic resonance imaging
LSI: Limb symmetry index
EPIC: Estimated pre-injury capacity
PROMs: Patient-reported outcome measures
